# Effects of season and experimental warming on the bacterial community in a temperate mountain forest soil assessed by 16S rRNA gene pyrosequencing

**DOI:** 10.1111/j.1574-6941.2012.01420.x

**Published:** 2012-06-25

**Authors:** Melanie Kuffner, Brigitte Hai, Thomas Rattei, Christelle Melodelima, Michael Schloter, Sophie Zechmeister-Boltenstern, Robert Jandl, Andreas Schindlbacher, Angela Sessitsch

**Affiliations:** 1 Bioresources Unit, Department of Health and Environment, AIT – Austrian Institute of Technology GmbHTulln, Austria; 2 Research Unit Environmental Genomics, German Research Center for Environmental Health, Helmholtz Zentrum MünchenNeuherberg, Germany; 3 Department of Computational Systems Biology, Ecology Centre, University of ViennaVienna, Austria; 4 Laboratoire d'Ecologie Alpine, CNRS UMR 5553, Université J. FourierGrenoble, France; 5 Department of Forest Ecology and Soils, Federal Research and Training Centre for Forests, Natural Hazards and Landscape (BFW)Vienna, Austria

**Keywords:** 454 pyrosequencing, soil bacterial community, climate change, ecological coherence, season, soil warming

## Abstract

Climate warming may induce shifts in soil microbial communities possibly altering the long-term carbon mineralization potential of soils. We assessed the response of the bacterial community in a forest soil to experimental soil warming (+4 °C) in the context of seasonal fluctuations. Three experimental plots were sampled in the fourth year of warming in summer and winter and compared to control plots by 16S rRNA gene pyrosequencing. We sequenced 17 308 amplicons per sample and analysed operational taxonomic units at genetic distances of 0.03, 0.10 and 0.25, with respective Good's coverages of 0.900, 0.977 and 0.998. Diversity indices did not differ between summer, winter, control or warmed samples. Summer and winter samples differed in community structure at a genetic distance of 0.25, corresponding approximately to phylum level. This was mainly because of an increase of *Actinobacteria* in winter. Abundance patterns of dominant taxa (> 0.06% of all reads) were analysed individually and revealed, that seasonal shifts were coherent among related phylogenetic groups. Seasonal community dynamics were subtle compared to the dynamics of soil respiration. Despite a pronounced respiration response to soil warming, we did not detect warming effects on community structure or composition. Fine-scale shifts may have been concealed by the considerable spatial variation.

## Introduction

The community of heterotrophic soil microorganisms is responsible for the mineralization of soil organic matter (SOM). A share of the consumed organic carbon leaves the soil as CO_2_ in a process referred to as heterotrophic soil respiration. Soil respiration has been predicted to accelerate under climate warming and thus to feed back on global temperature increase (Cox *et al*., 2000; Davidson & Janssens, 2006). Soil warming experiments (0.3–6 °C) in various types of ecosystems consistently produced an immediate increase in soil respiration (Rustad *et al*., 2001). However, the long-term development of carbon mineralization is uncertain, as other environmental factors such as substrate quality and water availability interfere with temperature effects. Particularly, depletion of readily decomposable SOM may reverse the initial respiration increase (Kirschbaum, 2004). Moreover, it has been postulated that climate change may induce shifts in the community composition of soil microorganisms (Bardgett *et al*., 2008). Warming may give competitive advantage to species adapted to higher temperatures (Rinnan *et al*., 2009), which are supposed to allocate more of a given carbon substrate into their biomass and less into respiration (Bradford *et al*., 2010), resulting in reduced CO_2_ emissions from soil (Allison *et al*., 2010). On the other hand, a new community constellation may facilitate decomposition of recalcitrant compounds, which constitute the bulk of organic matter stored in soils, and thus enhance CO_2_ efflux (Davidson & Janssens, 2006). Apart from direct effects on carbon cycling processes, community changes, particularly diversity losses, may reduce ecosystem resilience to future disturbances (Yachi & Loreau, 1999; Elshahed *et al*., 2008).

Contrasting effects of experimental warming on soil microbial community composition have been observed in different ecosystems (Rinnan *et al*., 2007, 2011; Cruz-Martinez *et al*., 2009; Castro *et al*., 2010; Yergeau *et al*., 2012). In Europe, forests are the main terrestrial carbon sinks, and warming-related developments in forest soils are of particular concern (Janssens *et al*., 2003). In mid-latitude forests, it is important to understand possible effects of warming in the context of seasonal temperature fluctuations, as soil respiration follows the annual course of soil temperature, moisture and substrate supply (Scott-Denton *et al*., 2006). In addition, different microbial taxa have been observed to reach their abundance maxima at different times of the year (Lipson, 2007; Kaiser *et al*., 2010). In this study, we investigated the effects of experimental soil warming on bacterial community composition in a temperate mountain forest soil. A field experiment has been established in 2004, with warmed plots where soil temperature was kept 4 °C above the temperature in control plots throughout each snow free season. A recent phospholipid fatty acid (PLFA) analysis has shown that 4 years of soil warming did not affect the abundance of fungi and bacteria or the ratio of Gram positive to Gram negative bacteria at this site (Schindlbacher *et al*., 2011). However, the phylogenetic resolution of the PLFA approach may have been too coarse to detect effects of warming. It has been suggested that different biological patterns can be observed at different phylogenetic levels (Philippot *et al*., 2010). Therefore, in the present study, we applied 16S rRNA gene pyrosequencing (454) for in-depth analysis of the bacterial community, which dominated over fungal populations in this soil (Schindlbacher *et al*., 2011). We analysed samples from July 2008 and from below the snow cover of March 2009 to focus on the effects of experimental warming (+4 °C) and on fluctuations in community composition between summer and winter. The aims of the study were (1) to assess the seasonal plasticity of the bacterial community, (2) to identify at which level of taxonomic resolution seasonal shifts can be observed best, (3) to determine whether experimental warming introduced additional shifts at the same or at any other taxonomic level, and (4) to differentiate whether possible warming-induced changes were transient and only detectable in summer, or persistent throughout the following winter.

## Materials and methods

### Study site and soil sampling

The study site is located 910 m a.s.l. in the North Tyrolean Limestone Alps near Achenkirch, Austria (11°38′221″ E; 47°34′50″ N). The 130-years-old mountain forest is dominated by Norway spruce (*Picea abies*) and interspread with European beech (*Fagus sylvatica*) and silver fir (*Abies alba*). Soils have been characterized as Chromic Cambisols and Rendzic Leptosols with deep A horizons and mull as dominant humus form. The bedrock is dolomite, and soil pH is slightly above 6. The climate at the site is cool and humid with average annual temperature and precipitation values of 5.7 °C and 1480 mm, respectively (1987–2007). The snow free season typically lasts from April/May until November. Three control plots and three warmed plots of 2 × 2 m were established in 2004. Resistance heating cables (Etherma, Salzburg) were placed into the soil at 3 cm depth with 7.5 cm distance between cable lines. Cables in the control plots were not heated. In the warmed plots, soil temperature at 5 cm depth was kept 4 °C above the temperature of the control plots during snow free seasons. During snow covered periods, warming was suspended because a continuous snow cover insulated soil temperatures from air temperatures. For a detailed description of the study site and the soil warming system, see Schindlbacher *et al*. (2007, 2009).

In the current study, we sampled on 29 July 2008 (summer samples) and on 18 March 2009 (winter samples). In March, samples were taken from below the snow cover. Two cores of approximately 100-g fresh mineral soil were taken from the top 5 cm of each plot. The cores were separately homogenized and aliquots of 2 g were immediately frozen in liquid nitrogen and stored at −80 °C.

### Soil DNA extraction, preparation of 16S rRNA gene amplicons and 454 pyrosequencing

DNA was obtained from 0.5 g of homogenized soil by phenol–chloroform extraction according to Griffiths *et al*. (2000) with modifications (Costa *et al*., 2004). DNA extracts were purified using custom Sepharose® (Sigma) CL6B columns. The two DNA samples from each plot were pooled in equimolar ratios. Template DNA concentration was chosen according to preliminary qPCR experiments. Approximately, 5 × 10^6^ 16S rRNA gene copies were used as template in 25 μL PCR reactions, containing 1× Fast start HiFi Polymerase Buffer, 0.05 U μL^−1^ Fast start HiFi Polymerase (Roche, Mannheim, Germany), 0.2 mM of each dNTP, 0.2 μM of each primer, 1 mM BSA and 2% DMSO. Cycling conditions were 27 cycles of 30 s denaturation at 94 °C, 45 s annealing at 68.5 °C and 90 s elongation at 72 °C, preceded by an initial denaturation step of 3 min at 94 °C, and terminated by a final elongation step of 7 min at 72 °C. The conserved bacterial 16S primers 27f (5′-AGAGTTTGATCCTGGCTCAG-3′; Weisburg *et al*., 1991) and 518r (5′-ATTACCGCGGCTGCTGG-3′; Muyzer *et al*., 1993) were used to amplify a fragment containing the variable regions V1, V2 and V3. At their 5′ ends, the primers carried either the 454-adaptor B or the 454-adaptor A with a specific 10 nt barcode for each soil sample (Supporting Information, Table S1). From each of the 12 DNA samples, two independent amplicon libraries were prepared in separate PCRs: One with adaptor A and barcode attached to 27f, to be sequenced from the V1 end, the other with adaptor A and barcode attached to 518r, to be sequenced from the V3 end of the amplicon. The PCR products were quantified using a Bioanalyzer 2100 (Agilent, Santa Clara, CA). Equimolar amounts of PCR products were pooled and purified with the AMPure purification system (Beckman Coulter, Danvers, MA). Pyrosequencing was carried out on a GS FLX machine (Roche, Mannheim, Germany), using titanium reagents and following the instructions of the manufacturer.

### Sequence data processing

Sequence processing was carried out in mothur (Schloss *et al*., 2009), except for quality filtering which was done in LUCY (Chou & Holmes, 2001), and the second preclustering step, which was done in CD-HIT-EST (Li & Godzik, 2006). Our procedure was the following: Reads lacking the initial barcode and primer sequence and reads in which LUCY did not identify a 100 nt window with an average error rate below 0.002 were discarded. The remaining 454 reads were aligned with the SILVA template alignment provided on the mothur platform using the Needleman pairwise alignment algorithm. Reads that did not span the stretch from *Escherichia coli* position 27–518 were removed. Overlapping reads differing by less than 1.5% of total residues were grouped by single linkage preclustering in mothur (Huse *et al*., 2010). Further preclustering was carried out in CD-HIT-EST to group reads differing by < 3% of total residues. One representative read of each precluster was checked with mothur's chimera.slayer, and preclusters of chimeric reads were eliminated. To obtain operational taxonomic units (OTUs), pairwise distances between sequences were calculated using mothur's ‘one gap’ scoring scheme, and clusters were built using the average neighbour algorithm recommended by Schloss & Westcott (2011). For phylogenetic identification, the 454 reads were compared to the SILVA bacterial 16S rRNA gene database using the Bayesian classifier implemented in mothur and a confidence threshold of 80% (bootstrap). The unprocessed sequence set was deposited in the NCBI Short Read Archive under the accession number SRP009837.

### Statistical analysis

To standardize samples, each of the 24 sequence libraries was subsampled to the size of the smallest library. Pairs of libraries obtained from the same soil DNA, but sequenced from the 27f and the 518r ends, respectively, were pooled. The abundance-based coverage estimator (ACE) and the inverse Simpson index (1/*D*) were calculated in mothur. The influence of experimental warming on diversity indices was evaluated separately for summer and winter sample sets (*n* = 3) using t-tests for independent samples in R! (http://www.R-project.org). As warming had no significant influence on diversity, controls and warmed samples were analysed together for differences between summer and winter in paired t-tests (*n* = 6). Phylotypes and OTUs comprising at least 120 reads, corresponding to an average of 10 reads per sample, were individually analysed for abundance shifts related to season and warming. To identify significant seasonal shifts, we screened the sets of abundant phylotypes and OTUs (≥ 120 reads) for individuals that either increased or decreased from summer to winter in each of the six plots. Individuals with such consistent summer-winter shifts were checked for a possible influence of experimental warming on relative abundance using *t*-tests for independent samples. As no significant warming effects were detected, the significance of the seasonal shifts was evaluated taking controls and warmed samples together (*n* = 6) and using paired *t*-tests. *P*-values were fdr-corrected for multiple comparisons. To search the sets of abundant phylotypes and OTUs for taxa with abundance shifts related to warming, we screened for individuals that were on average at least 1.5 times more or less abundant in warmed plots compared to controls. Separate screenings were carried out with summer and winter samples, and the significance of the observed shifts was evaluated in *t*-tests for independent samples (*n* = 3) followed by fdr-correction. Prior to each *t*-test for independent samples, normal distribution and variance homogeneity of abundances were evaluated by Shapiro–Wilk and Levene tests, respectively. Prior to each paired *t*-test, normal distribution of the pairwise differences was checked in a Shapiro–Wilk test. Phylotypes and OTUs with left-skewed abundance distributions were square root–transformed. Taxa lacking normal distribution after square root transformation were excluded from statistic analysis. Rarefaction analysis, nonmetric multidimensional scaling (NMDS; Faith *et al*., 1987) and nonparametric multivariate analysis of variance (npmanova; Anderson, 2001) were carried out in R!, using the Vegan package (Oksanen *et al*., 2007). Pairwise dissimilarities between individual soil sample communities were calculated based on the abundance patterns of OTUs and phylotypes using Vegan's abundance variant of the Jaccard index. Alternatively, pairwise dissimilarities were calculated using the weighted Unifrac metric (Lozupone & Knight, 2005) based on a relaxed neighbour-joining tree constructed in clearcut (Evans *et al*., 2006). The dissimilarities were ordinated in two-dimensional NMDS plots, and the significance of seasonal and warming-related changes in community structure was evaluated by two-way npmanova. npmanova has proven to be suitable for testing the effects of environmental factors on complex microbial communities in soil (Lentendu *et al*., 2011).

## Results

### General characteristics of the 454 sequence data set

We analysed bacterial 16S rRNA gene amplicons prepared from 12 forest soil DNA samples, originating from three warmed plots and three control plots sampled in summer and winter. Bacterial biomass was, according to soil levels of bacterial PLFAs, similar in summer and winter and not detectably affected by warming. Soil respiration was about 20 times higher in summer and increased by another 16–34% in warmed plots (Table 1). Two independent libraries of amplicons spanning *E. coli* positions 27 through 518 were prepared from each DNA sample and sequenced from the primers 27f and 518r, respectively (Table S1). This was carried out to enable community analysis based on V1–V2 as well as V3 sequence information even in the case of short final read lengths. Eventually, 454 pyrosequencing produced reads spanning the entire PCR amplicons (*E. coli* positions 27–518). In total, 260 592 nonchimeric, high quality, whole-amplicon reads were obtained. All 24 libraries were subsampled to 8654 reads each, which was the size of the smallest library (Table S1). Libraries sequenced from the 518r side contained more reads affiliated with *Actinobacteria* and *Deltaproteobacteria* and less *Gammaproteobacteria*, *Chloroflexi*, *Planctomyctetes* and unclassified reads than libraries sequenced from the 27f side (Fig. S2). These effects were constant across all samples and most likely due to the fact that only primers at read start carried 10 nt barcodes, which may have influenced the annealing selectivity (Berry *et al*., 2011). To present a larger share of the soil bacterial community, we merged pairs of complementary oriented libraries yielding one pooled library of 17 308 reads per soil sample. All results presented in this article were obtained with the 12 pooled libraries. Diversity characteristics of the 24 individual libraries are presented in Fig. S3.

**Table 1:** Soil characteristics in summer and winter

Sample (symbol used in figures)	Plot	Treatment	Soil temperature (°C)	Bacterial PLFA* (mmol g^−1^ DW)	Soil respiration* (μmol m^−2^ s^−1^)
Summer (29 July 2008)					
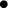	1	Control	13.0	209	4.47 (± 0.19)
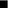	2	Control	13.4	136	5.69 (± 0.62)
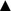	3	Control	13.4	144	4.19 (± 0.37)
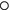	4	Warmed	16.8	158	5.20 (± 0.63)
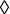	5	Warmed	17.3	132	6.67 (± 1.73)
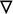	6	Warmed	16.7	128	5.60 (± 0.56)
Winter (18.03.2009)					
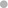	1	Control	0.6	228	0.22 (± 0.03)
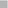	2	Control	ND	134	0.26 (± 0.01)
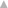	3	Control	ND	199	0.24 (± 0.01)
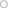	4	Warmed†	0.6	231	0.13 (± 0.03)
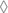	5	Warmed†	ND	166	0.33 (± 0.03)
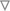	6	Warmed†	ND	166	0.20 (± 0.02)

* Data from Schindlbacher *et al*., 2011.

† The same plots were warmed every snow free season, warming was suspended every winter.

### Diversity of OTUs

**Fig. 1:**
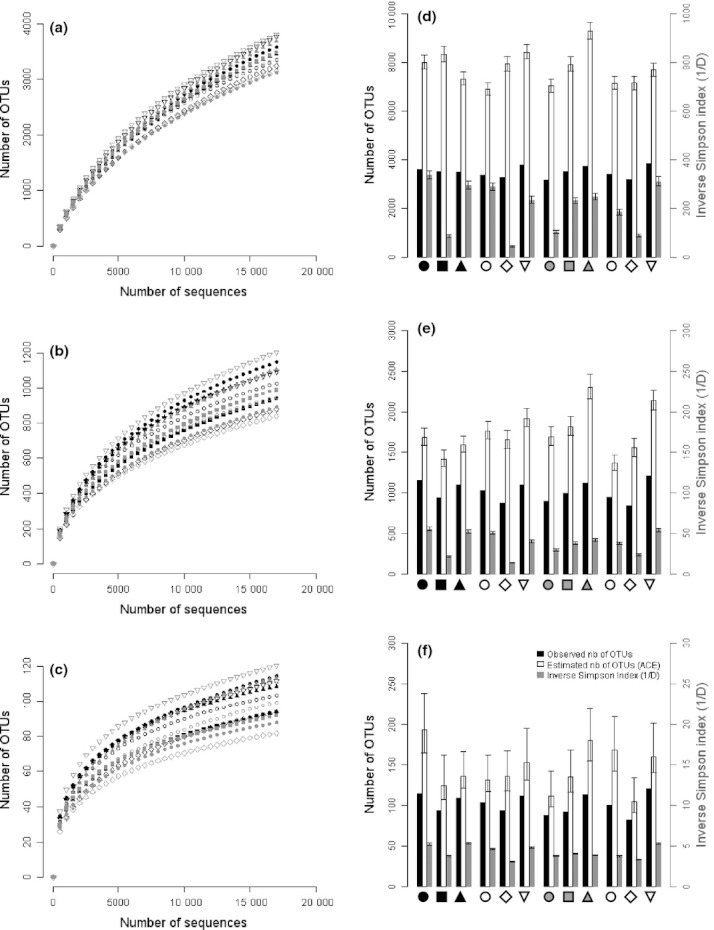
Diversity of OTUs. Rarefaction analysis, OTU counts and estimates of richness and diversity. (a and d) OTUs_0.03_, (b and e) OTUs_0.10_, (c and f) OTUs_0.25_. Sample symbols: • ▪ ▲ – control plots, ○ ◊ ▽ – warmed plots, black-summer, grey-winter. In panels d–f observed OTUs, and ACEs are plotted on the primary, 1/*D*-values on the secondary *y*-axis. Error bars span the 95% confidence intervals.

We analysed OTUs at genetic distances of 0.03 (OTUs_0.03_), 0.10 (OTUs_0.10_) and 0.25 (OTUs_0.25_) corresponding to average intra-OTU sequence identities of 97%, 90% and 75%, respectively. 0.03 is the narrowest clustering distance recommended for 454 sequences (Kunin *et al*., 2010), and we found 0.25 to be the largest distance appropriate for the present data set. At higher clustering distances, the proportion of rare OTUs increased signalizing fusions of large OTUs rather than integration of rare OTUs into bigger clusters (Fig. S1). We chose 0.10 as an intermediate level of genetic distance. Total numbers of OTUs_0.03_, OTUs_0.10_ and OTUs_0.25_ in the data set were 14 976, 3381 and 238, respectively. Per individual sample, we observed on average 3491 OTUs_0.03_, 1020 OTUs_0.10_ and 102 OTUs_0.25_ (Fig. 1) with corresponding average Good's coverages of 0.900, 0.877 and 0.998, respectively. All rarefaction curves were unsaturated and sustained crossing suggested that 17 308 reads per sample were insufficient to capture the final richness ranking among samples even at a genetic distance of 0.25. To estimate this final ranking, we calculated ACEs. Furthermore, we compared sample diversities based on the inverse Simpson index (1/*D*), which integrates evenness information. Confidence intervals (95%) around ACEs and 1/*D*-indices (error bars in Fig. 1d–f) and around rare-faction curves (not shown) suggested that differences between individual soil samples were significant. However, these differences were not related to season or warming (*t*-test, *P* > 0.05). The fact that diversity rankings differed between genetic distances as well as between ACE and 1/*D* at each distance, reflects differences between the 12 samples in community structure.

### Community structure based on OTUs

**Fig. 2:**
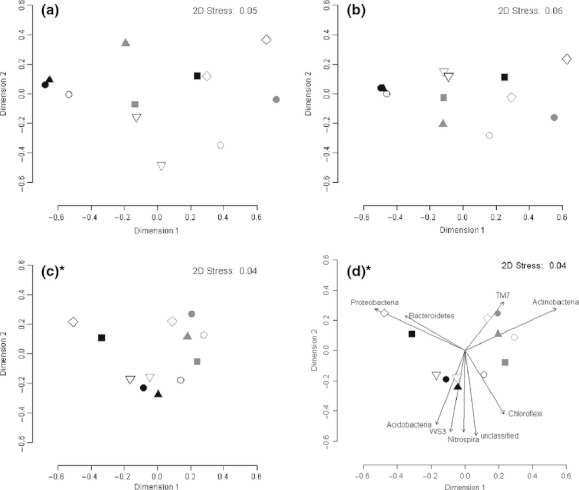
Differences in community structure. NMDS of pairwise Jaccard distances between samples. The abundance variant of the Jaccard index was calculated based on the distribution of (a) OTUs_0.03_, (b) OTUs_0.10_, (c) OTUs_0.25_ and (d) phylum affiliations of the individual reads. Arrows indicate the direction of increased abundance of phyla, for which a significant gradient is displayed in the NMDS (*P* < 0.1). Sample symbols: • ▪ ▲ – control plots, ○ ◊ ▽ – warmed plots, black-summer, grey-winter. *Significant difference between summer and winter (npmanova, *P* < 0.05).

To visualize overall similarities and differences in community structure between the 12 samples, pairwise Jaccard dissimilarities were calculated from OTU abundance patterns and ordinated in two-dimensional NMDS plots (Fig. 2a–c). At a genetic distance of 0.03, no clustering related to season or warming occurred. At a genetic distance of 0.25, a separation of summer and winter samples was observed, and npmanova confirmed that OTU composition differed significantly between summer and winter (*P* < 0.05). NMDS at a genetic distance of 0.10 produced an intermediate between the pictures obtained at 0.03 and 0.25, without significant separation of summer and winter samples. The same NMDS was carried out at all genetic distances between 0.03 and 0.30 (data not shown). No grouping of samples related to soil warming was observed at any distance, and seasonal effects were significant only at genetic distances between 0.22 and 0.25 (*P* < 0.05). The weighted Unifrac distance metric, which integrates all possible taxonomic levels, produced an NMDS intermediate to that of OTUs_0.10_ and OTUs_0.25_ and revealed no significant difference between summer and winter (Fig. S4).

### Phylotype composition

**Fig. 3:**
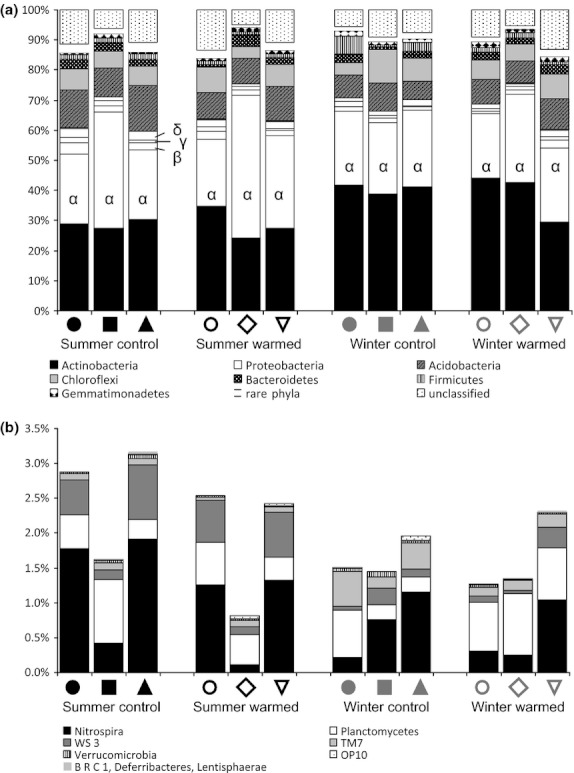
Phylogenetic community composition. Relative abundance of (a) abundant phyla (> 1% of total reads) and proteobacterial classes, (b) rare phyla. Sample symbols: • ▪ ▲ – control plots, ○ ◊ ▽ – warmed plots, black – summer, grey – winter.

In addition to OTU-based analysis, we assigned the individual reads to phylotypes by sequence comparison to the SILVA 16S rRNA gene database. At all taxonomic levels, phylotypes containing more than 120 reads were considered sufficiently abundant for statistic analysis of their distribution among soil samples. This criterion was met at the highest taxonomic level by 11 phyla representing 91% of all reads, and at the lowest level by 43 genera representing 34% of all reads. Table 2 lists all phylotypes identified at any taxonomic level, which either increased or decreased from summer to winter consistently in all soil plots. We observed a significant increase in the proportion of *Actinobacteria* (paired *t*-test, *P* < 0.05), which was compensated by a tendentious decrease of *Acidobacteria* and *Proteobacteria* (Table 2). Even so, the overall phylum abundance pattern was relatively stable across all samples (Fig. 3), suggesting temporal stability also at finer taxonomic scales. However, several of these shifts were statistically significant (paired *t*-test, *P* < 0.05), and seasonal dynamics appeared to be coherent within taxonomic lineages: Decreases from summer to winter were only detected in members of the phyla *Acidobacteria* and *Proteobacteria*, whereas increases were only detected in members of *Actinobacteria*, TM7-Bacteria and *Chloroflexi*. To assess overall similarities and differences between the phylum abundance profiles of the 12 samples, we applied the same NMDS approach that we had used for OTUs. The resulting graph (Fig. 3). Below phylum level, only a small number of phylotypes showed consistent seasonal shifts in all plots (Table 2d) was very similar to that obtained from OTUs_0.25_ (Fig. 2c) and npmanova confirmed a significant difference between summer and winter profiles (*P* < 0.05). Projecting the abundance variation of the most dynamic phyla into the NMDS confirmed that summer and winter samples mainly differed in the abundance of *Actinobacteria*, *Acidobacteria* and *Proteobacteria*. The isolated position of summer samples from plots 2 and 5 was attributable to their exceptionally high content in *Proteobacteria* (compare Figs 2d and 3a). Significant warming effects on the abundance of individual phylotypes were not detected.

**Table 2:** Phylotypes with abundance shifts between summer and winter in all plots

	Relative abundance (reads per sample)†	
	
Phylotype§	Summer	Winter
*Increased relative abundance in summer*		
*Acidobacteria*‡	1914 (±182)	1417 (±107)
Group 7*	79 (±5)	50 (±10)
Group 3	22 (±5)	9 (±1)
*Proteobacteria* (*Rhizobiales* (α*-Proteobacteria*))¶	6369 (±637)	5009 (±196)
*Rhodobiaceae*	83 (±20)	31 (±10)
Hyphomicrobiaceae*	1792 (±50)	1120 (±83)
*Pedomicrobium*‡*	201 (±17)	98 (±20)
Rhodoplanes*	1050 (±98)	829 (±53)
Unclassified Hyphomicrobiaceae*	303 (±70)	91 (±14)
Phyllobacteriaceae*	194 (±18)	110 (±28)
*Increased relative abundance in winter*		
Actinobacteria*	4998 (±253)	6872 (±375)
(*Acidimicrobiales*)¶		
Acidimicrobiaceae*	198 (±10)	270 (±24)
Solirubrobacterales*	2180 (±195)	3030 (±225)
*Conexibacteraceae*	1543 (±164)	2081 (±163)
Solirubrobacteraceae*	398 (±14)	628 (±70)
TM7	15 (±1)	44 (±11)
(*Chloroflexi*)¶		
Unclassified Thermomicrobia*	27 (±3)	42 (±6)

* Significant difference between summer and winter (paired *t*-test, *n* = 6, *P* < 0.05).

† In total, 17 308 reads were analysed from each sample, so that the number of reads affiliated with a given phylotype corresponds to the relative abundance of this phylotype. Means (±SE) are given and were calculated over the six samples obtained from the six different plots.

‡ Data were square root–transformed for the paired *t*-test.

¶ For easier perception of phylogenetic lineages, additional taxonomic levels, which did not display seasonal shifts, are included between parentheses.

§ Out of all identified phylotypes at any taxonomic level, the table contains those that consistently either increased or decreased from summer to winter in all plots.

### Relative abundance of dominant OTUs

Phylotyping only addressed the fraction of sequences that were related to identified taxa and may have been inaccurate in lineages with few representatives in the database. Therefore, we also analysed the distribution of OTUs, to verify the observed patterns of coherent seasonal dynamics among related taxa. We evaluated seasonal and warming-related abundance shifts at each genetic distance in OTUs containing more than 120 reads. This criterion was met by 251 OTUs_0.03_ representing 50% of all reads, by 214 OTUs_0.10_ representing 86% and by 38 OTUs_0.25_ representing 99% of all reads (Fig. S1D). Eighteen OTUs_0.03_, twelve OTUs_0.10_ and two OTUs_0.25_ displayed significant abundance changes between summer and winter (Table S2). To compare the results obtained with OTUs to results of the phylotype analysis, we considered the consensus affiliation of each OTU. At each genetic distance, OTU abundance distributions confirmed the previously detected seasonal coherence within lineages. The majority of OTUs showing significant seasonal shifts (Table S2) were members of the taxa listed in Table 2. Opposed seasonality of OTUs within one phylum was not observed. Significant effects of experimental warming on the relative abundance of individual OTUs were not detected at any genetic distance. OTUs_0.10_ were most suitable for confirming and further resolving the abundance patterns detected in phylotype analysis. The genetic distance of 0.25 effectively corresponded to phylum level and provided little additional information. Most known phyla were represented by 1–3 large OTUs_0.25_, and unclassified reads clustered in one large and multiple rare OTUs_0.25_. At a genetic distance of 0.03, half of all reads including 76% of the acidobacterial sequences fell into OTUs_0.03_ smaller than 120 reads. For comparison to other studies, Table S3 lists the top 30 OTUs_0.03_.

## Discussion

Deep sequencing has enabled us to thoroughly characterize the bacterial community of a temperate mountain forest soil. The analysed community showed the typical set of dominant soil phyla (Janssen, 2006), with an exceptionally high proportion of *Actinobacteria* and a low proportion of *Acidobacteria* compared to the global average of soil communities (Lauber *et al*., 2009). We note that being a PCR-based method, pyrosequencing does not produce quantitative data. Relative sequence abundances in the amplicon libraries may not reflect actual ratios between taxa in the soil. In our study, pooling of libraries affected by two different barcoding biases probably led to additional distortion. Therefore we will focus on comparisons between the 12 – equally biased – libraries, rather than discussing the composition of individual samples. OTU diversities observed here cannot be compared directly to most previous 454-based soil studies, which have been carried out with different primers. The observation that 17 308 reads were insufficient to cover the diversity of OTUs_0.03_, OTUs_0.10_ and even OTUs_0.25_ corresponds to the findings of Roesch *et al*. (2007). These authors estimated that over 100 000 reads may be required to detect all OTUs_0.20_ in a soil. However, for a relative comparison of diversity levels between samples, exhaustive sequencing is not required (Shaw *et al*., 2008).

To capture the annual amplitude of climatic variation at the study site, we analysed samples from mid-summer (July 29) and late winter (March 18). Soil temperature had fluctuated between 10 °C and 15 °C (14–19 °C in warmed plots) for 2 months prior to the summer sampling and had been slightly above 0 °C under the snow cover for 4 months prior to winter sampling. Soil moisture was in a similar range prior summer and winter sampling on all plots (Schindlbacher *et al*., 2012). Microbial activity was strongly correlated with soil temperature (Schindlbacher *et al*., 2011), and soil respiration was almost 20 times higher during summer sampling than during winter sampling (Table 1). However, bacterial biomass did not decrease from summer to winter (Table 1), and the results of this amplicon sequencing study suggest that also the community composition of soil bacteria was relatively stable. We observed moderate shifts in the relative abundances of individual groups but no switches between dominant and rare taxa. Similarly, previous surveys on seasonal dynamics in alpine and agricultural soils reported rather subtle shifts in the course of the year (Lipson, 2007; Zinger *et al*., 2009; Inceoglu *et al*., 2011). Moreover, a recent study on seasonal dynamics in forest soils highlighted that seasonal changes in CO_2_ efflux cannot be directly related to shifts in the bacterial community structure (Chemidlin Prevost-Boure *et al*., 2011). We note that only a subset of all soil bacteria is active at any moment (Prosser *et al*., 2007). Therefore, differences between active summer and winter populations may have been incompletely reflected in total community DNA. The only available pyrosequencing study comparing active and total bacterial communities in a forest soil documents limited overlapping of DNA- and RNA-derived sequence libraries (Baldrian *et al*., 2012).

The observed seasonal shifts resembled those detected in conventional clone libraries from alpine and subalpine soils (Lipson, 2007), particularly the increase of *Actinobacteria* in winter and the increase of certain *Proteobacteria* in summer. Lipson (2007) interpreted seasonal maxima of different phyla as reactions to fluctuations in carbon substrate supply from root exudation and plant litter. In fact, short and medium term impacts of substrate quality and availability on soil bacterial communities have been experimentally demonstrated (Strickland *et al*., 2009). It is conceivable that the seasonal shifts in bacterial community composition at our study site were related to dynamics in substrate supply. The summer sampling in July fell into a period of high root exudation, if we consider root respiration levels of previous years (Schindlbacher *et al*., 2009) as indicator for general root activity. Moreover, reduced substrate availability was observed in winter (A. Schindlbacher, unpublished data). The direct influence of seasonal variations in soil temperature on microbial population dynamics is not well documented. Available information refers to extreme conditions (Waldrop & Firestone, 2006; Haei *et al*., 2010) rather than to fluctuations within the annual temperature spectrum.

An essential observation of this survey was the coherence of seasonal fluctuations within phylogenetic lineages, which was consistently detected in OTU-based and phylotype-based analysis (Table 2 and Table S2). Abundance shifts of related taxa accumulated with increasing taxonomic rank and overall differences in community structure appeared only at phylum level or at an OTU distance of 0.25 (Fig. 2). Coherence of high-level soil taxa in their biogeography (Barberan *et al*., 2012), and in their response to environmental factors such as carbon source availability (Fierer *et al*., 2007), pH (Lauber *et al*., 2009) or agricultural management (Philippot *et al*., 2009), is a recently discovered phenomenon. This so-called ‘ecological coherence’ does not imply that taxon-specific traits are uniformly shared by all taxon-members. However, it may reflect the role of ecological adaptation as driving force in the evolution of deep branches in bacterial phylogeny (Philippot *et al*., 2010). Coherent seasonal shifts within bacterial lineages have been described previously in aquatic bacterioplankton (Eiler *et al*., 2012).

The seasonal shifts in phylum abundance patterns observed here were not accompanied by detectable changes in diversity. This is plausible, as we have demonstrated multiple members of each phylum to undergo synchronous seasonal fluctuations. Moreover, Lauber *et al*. (2009) have reported that in many soils, intra-phylum diversity is similar across phyla. Independent variation of soil community composition and diversity has been observed previously (Ge *et al*., 2010; Ramirez *et al*., 2010), suggesting that these two community parameters may be controlled by different factors. In our forest site, diversity varied between soil samples that were taken only few metres apart from one another. Strong seasonal variations of bacterial diversity have been detected in an agricultural soil, which was spatially homogenized and where the course of the year was determined by the synchronized development of annual crop plants (Inceoglu *et al*., 2011). In temperate forest soils, inter-annual community variations have been observed in addition to spatial heterogeneity (Rasche *et al*., 2010). Therefore, it would be interesting to extend both spatial and temporal replication in future studies.

Although experimental warming by four degrees enhanced soil respiration by 16–34% (Table 1), we did not detect changes in bacterial community composition, structure or diversity. Given the subtle response of the bacterial community to seasonal temperature variations of 13–17 °C, it is reasonable that 4 °C of warming did not provoke immediate shifts within the detection range of this study. Long-term effects of warming may emerge only after more than a decade and have been associated with warming-induced vegetation changes or changes in substrate availability (Rinnan *et al*., 2007). At our forest site, 4 years of warming did not alter vegetation, nor did it lead to a depletion of decomposable substrates (Schindlbacher *et al*., 2011). Rapid community changes have been observed in particularly vulnerable Antarctic soils (Yergeau *et al*., 2012) and in temperate soils upon exposure to temperature maxima exceeding the local ‘climate history’ (Waldrop & Firestone, 2006). In our experimental plots, temperature maxima were within the historical range. Temperature minima in winter, which likewise exert selective force on soil bacteria (Rinnan *et al*., 2009), were near 0 °C in both warmed plots and controls. Warmed plots were not manipulated in winter, as the site is typically covered in deep snow from November to April (Schindlbacher *et al*., 2007), and changes in winter temperatures are unlikely as long as a continuous winter snow cover is preserved. Finally, we emphasize that, although triplicates were ideal for soil respiration monitoring and PLFA analysis (Schindlbacher *et al*., 2011), additional plots and higher sampling density within plots would have been required to better account for the spatial heterogeneity of the bacterial community DNA. The limited number of available replicate plots precluded the detection of warming-induced population shifts on a fine scale. Our results suggest that experimental warming during four snow free seasons did not induce prominent changes in the bacterial community composition at DNA level. As bacterial biomass did not change significantly either (Table 1), the respiration response obviously reflected an increase in bacterial activity. RNA or protein-level data would have helped linking the rise in respiration to increased activity in specific taxa or mineralization pathways.

In conclusion, the merit of analysing over 17 000 reads per sample was our ability to document coherence of seasonal fluctuations among related taxa at various levels of resolution. Owing to this coherent behaviour of lower-level taxa, overall differences between summer and winter communities were most clearly discernable at the phylum level and at an OTU distance of 0.25. Analysing OTUs at a genetic distance of 0.10 provided details on individual taxa involved in the seasonal shifts. In contrast, the genetic distance of 0.03 dissected a large share of the sequence set into very rare OTUs, for which detection in individual samples depended probably on chance even at the present sequencing depth (Roesch *et al*., 2007). For a mere detection of the seasonal shifts, fewer reads per sample would have been sufficient. Sensitivity for possible fine scale effects of experimental warming could be improved by analysing additional replicate plots or time points. Therefore, we conclude in accordance with the simulation results reported by Kuczynski *et al*. (2010) that future studies on soil community fluctuations should exploit the power of 454 sequencing to extend replication rather than sequencing depth. The present data suggest that pronounced respiration responses to seasonal and experimental temperature changes occurred in a relatively stable population of soil bacteria.

## Statement

Re-use of this article is permitted in accordance with the Terms and Conditions set out at http://wileyonlinelibrary.com/onlineopen#OnlineOpen_Terms.

## Supporting Information

**Fig. S1.** Clustering of reads into OTUs at different genetic distances.

**Fig. S2.** Phylogenetic community composition of libraries obtained with primers 27f and 518r at read start.

**Fig. S3.** OTU counts and estimates of richness and diversity of the 24 individual amplicon libraries.

**Fig. S4.** NMDS of weighted Unifrac distances between samples.

**Table S1.** List of soil samples and barcoded libraries.

**Table S2.** (A) OTUs_0.03_ with abundance shifts between summer and winter in all plots. (B) OTUs_0.10_ with abundance shifts between summer and winter in all plots. (C) OTUs_0.25_ with significant abundance shifts between summer and winter in all plots.

**Table S3.** OTUs_0.03_ containing more than 600 reads ranked by their abundance.
