# Supplementary material for: Effects of season and experimental warming on the bacterial community in a temperate mountain forest soil assessed by 16S rRNA gene pyrosequencing
Source: FEMS Microbiol Ecol. 2012 Jun 25;82(3):551–62. doi: 10.1111/j.1574-6941.2012.01420.x (PMC3556523; doi:10.1111/j.1574-6941.2012.01420.x)
Supplement: Supplementary file 1 [file fem0082-0551-SD1.doc]

Table S1. List of soil samples and barcoded libraries

| **Sample** | | | | **MID** | | **16S Primer at read start** | **Nb. of** |
| --- | --- | --- | --- | --- | --- | --- | --- |
|  | Season | Warming | Plot | MID#^a^ | Sequence 5'-3' | Sequence 5'-3' | **qual. reads^b^** |
| *primer 27f at read start^c^* | | | |  |  |  |  |
| ● | summer | control | pl.1 | MID1 | ACGAGTGCGT | AGAGTTTGATCCTGGCTCAG | 9835 |
| ■ | summer | control | pl.2 | MID3 | AGACGCACTC | AGAGTTTGATCCTGGCTCAG | 9682 |
| ▲ | summer | control | pl.3 | MID5 | ATCAGACACG | AGAGTTTGATCCTGGCTCAG | 8728 |
| ○ | summer | warmed | pl.4 | MID2 | ACGCTCGACA | AGAGTTTGATCCTGGCTCAG | 11093 |
| **◊** | summer | warmed | pl.5 | MID4 | AGCACTGTAG | AGAGTTTGATCCTGGCTCAG | 16322 |
|  | summer | warmed | pl.6 | MID6 | ATATCGCGAG | AGAGTTTGATCCTGGCTCAG | 9968 |
| ● | winter | control | pl.1 | MID7 | CGTGTCTCTA | AGAGTTTGATCCTGGCTCAG | 10308 |
| ■ | winter | control | pl.2 | MID10 | TCTCTATGCG | AGAGTTTGATCCTGGCTCAG | 9113 |
| ▲ | winter | control | pl.3 | MID13 | CATAGTAGTG | AGAGTTTGATCCTGGCTCAG | 9813 |
| ○ | winter | warmed | pl.4 | MID8 | CTCGCGTGTC | AGAGTTTGATCCTGGCTCAG | 8654 |
| ◊ | winter | warmed | pl.5 | MID11 | TGATACGTCT | AGAGTTTGATCCTGGCTCAG | 11158 |
|  | winter | warmed | pl.6 | MID14 | CGAGAGATAC | AGAGTTTGATCCTGGCTCAG | 11010 |
| *Primer 518r at read start^c^* | | | |  |  |  |  |
| ● | summer | control | pl.1 | MID1 | ACGAGTGCGT | ATTACCGCGGCTGCTGG | 12421 |
| ■ | summer | control | pl.2 | MID3 | AGACGCACTC | ATTACCGCGGCTGCTGG | 14470 |
| ▲ | summer | control | pl.3 | MID5 | ATCAGACACG | ATTACCGCGGCTGCTGG | 9971 |
| ○ | summer | warmed | pl.4 | MID2 | ACGCTCGACA | ATTACCGCGGCTGCTGG | 12720 |
| ◊ | summer | warmed | pl.5 | MID4 | AGCACTGTAG | ATTACCGCGGCTGCTGG | 12139 |
|  | summer | warmed | pl.6 | MID6 | ATATCGCGAG | ATTACCGCGGCTGCTGG | 11132 |
| ● | winter | control | pl.1 | MID7 | CGTGTCTCTA | ATTACCGCGGCTGCTGG | 9172 |
| ■ | winter | control | pl.2 | MID10 | TCTCTATGCG | ATTACCGCGGCTGCTGG | 11208 |
| ▲ | winter | control | pl.3 | MID13 | CATAGTAGTG | ATTACCGCGGCTGCTGG | 10140 |
| ○ | winter | warmed | pl.4 | MID8 | CTCGCGTGTC | ATTACCGCGGCTGCTGG | 10225 |
| □ | winter | warmed | pl.5 | MID11 | TGATACGTCT | ATTACCGCGGCTGCTGG | 10609 |
|  | winter | warmed | pl.6 | MID14 | CGAGAGATAC | ATTACCGCGGCTGCTGG | 10700 |

^a^MID, multiplex identifyer = barcode, MID number from the 454-catalogue and barcode sequences are given. ^b^Number of non-chimeric high quality reads spanning the entire amplicon obtained in each library. Prior to statistical analysis all libraries were subsampled to 8654 reads, which was the size of the smallest library. ^c^From each soil DNA sample two barcoded amplicon libraries were prepared, one to be sequenced with primer 27f at read start and one to be sequenced with the primer 518r at read start.

**Table S2A: OTUs_0.03_ with abundance shifts between summer and winter in all plots**

| **OTU#^a^** | **Consensus affiliation^b^** | |  | **Relative abundance (reads per sample)^c^** | | | | |
| --- | --- | --- | --- | --- | --- | --- | --- | --- |
|  | **Phylum/class** | **Order/group** | **Family** | **Genus** | **Summer** | | **Winter** | |
| Increased relative abundance in summer | | |  |  |  |  |  |  |
| 476* | *Alphaproteobacteria* | *Rhizobiales* | *Hyphomicrobiaceae* | *Pedomicrobium* | 147 | (±17) | 65 | (±13) |
| 4543 | *Alphaproteobacteria* | *Rhizobiales* | *Hyphomicrobiaceae* | *Rhodoplanes* | 465 | (±25) | 340 | (±44) |
| 4620* | *Alphaproteobacteria* | *Rhizobiales* | *Hyphomicrobiaceae* | *Rhodoplanes* | 65 | (±10) | 26 | (±6) |
| 430* | *Alphaproteobacteria* | *Rhizobiales* | *Hyphomicrobiaceae* | *unclassified* | 44 | (±9) | 14 | (±5) |
| 590* | *Alphaproteobacteria* | *Rhizobiales* | *Hyphomicrobiaceae* | *unclassified* | 55 | (±12) | 16 | (±5) |
| 624* | *Alphaproteobacteria* | *Rhizobiales* | *Hyphomicrobiaceae* | *unclassified* | 14 | (±2) | 6 | (±2) |
| 327* | *Alphaproteobacteria* | *Rhizobiales* | *Rhodobiaceae* | *uncl.Rhodobiaceae* | 17 | (±5) | 4 | (±3) |
| 828* | *Alphaproteobacteria* | *Rhizobiales* | *Rhodobiaceae* | *uncl.Rhodobiaceae* | 21 | (±4) | 7 | (±2) |
| 551* | *Alphaproteobacteria* | *Rhizobiales* | *unclassified* | *unclassified* | 20 | (±5) | 5 | (±3) |
| 705* | *Alphaproteobacteria* | *Rhizobiales* | *unclassified* | *unclassified* | 52 | (±8) | 12 | (±4) |
| *Increased relative abundance in winter* | | |  |  |  |  |  |  |
| 7314^ND^ | *Actinobacteria* | *Acidimicrobiales* | *Acidimicrobiaceae* | *uncl.Acidimicrobiaceae* | 10 | (±2) | 17 | (±4) |
| 9372* | *Actinobacteria* | *Acidimicrobidae* | *Acidimicrobidae.inc.sedis* | *Ilumatobacter* | 7 | (±2) | 26 | (±5) |
| 10596* | *Actinobacteria* | *Actinobacteridae* | *Actinomycetales* | *Nocardioidaceae* | 14 | (±2) | 28 | (±4) |
| 10537^d^* | *Actinobacteria* | *Actinobacteridae* | *Actinomycetales* | *various* | 4 | (±2) | 25 | (±10) |
| 7878* | *Actinobacteria* | *Solirubrobacterales* | *Conexibacteraceae* | *Conexibacter* | 15 | (±4) | 30 | (±6) |
| 7926* | *Actinobacteria* | *Solirubrobacterales* | *Conexibacteraceae* | *Conexibacter* | 37 | (±15) | 91 | (±16) |
| 7975* | *Actinobacteria* | *Solirubrobacterales* | *Conexibacteraceae* | *Conexibacter* | 16 | (±3) | 46 | (±10) |
| 9323^ND^* | *Actinobacteria* | *unclassified* | *unclassified* | *unclassified* | 11 | (±3) | 41 | (±14) |
| 14089* | *Chloroflexi* | *Anaerolineales* | *Anaerolineaceae* | *uncl.Anaerolineaceae* | 10 | (±4) | 30 | (±10) |
| 14100^*^ | *Chloroflexi* | *Anaerolineales* | *Anaerolineaceae* | *uncl.Anaerolineaceae* | 9 | (±3) | 25 | (±8) |
| 11804* | *Chloroflexi* | *unclassified* | *unclassified* | *unclassified* | 206 | (±26) | 279 | (±46) |

**Table S2B: OTUs_0.10_ with abundance shifts between summer and winter in all plots**

| **OTU#^a^** | **Consensus affiliation^b^** | |  | **Relative abundance (reads per sample)^c^** | | | | |
| --- | --- | --- | --- | --- | --- | --- | --- | --- |
|  | **Phylum/class** | **Order/group** | **Family** | **Genus** | **Summer** | | **Winter** | |
| *Increased relative abundance in summer* | | |  |  |  |  |  |  |
| 114* | *Alphaproteobacteria* | *Rhizobiales* | *Hyphomicrobiaceae* | *various* | 503 | (±48) | 212 | (±47) |
| 112* | *Alphaproteobacteria* | *Rhizobiales* | *Hyphomicrobiaceae* | *uncl.Hyphomicrobiaceae* | 303 | (±69) | 91 | (±14) |
| 119* | *Alphaproteobacteria* | *Rhizobiales* | *Rhodobiaceae* | *uncl.Rhodobiaceae* | 83 | (±20) | 31 | (±10) |
| 74** | *Alphaproteobacteria* | *Rhizobiales* | *unclassified* | *unclassified* | 68 | (±7) | 18 | (±5) |
| 279* | *Alphaproteobacteria* | *various* | *various* | *various* | 128 | (±15) | 76 | (±15) |
| 1417 | *Betaproteobacteria* | *Burkholderiales* | *various* | *various* | 39 | (±11) | 16 | (±3) |
| 858^d^* | *unclassified* | *unclassified* | *unclassified* | *unclassified* | 18 | (±8) | 2 | (±1) |
| *Increased relative abundance in winter* | | |  |  |  |  |  |  |
| 1825* | *Actinobacteria* | *Acidimicrobiales* | *Acidimicrobiaceae* | *uncl.Acidimicrobiaceae* | 181 | (±9) | 253 | (±22) |
| 1731 | *Actinobacteria* | *Actinomycetales* | *Microbacteriaceae* | *various* | 85 | (±23) | 186 | (±29) |
| 81* | *Actinobacteria* | *Solirubrobacterales* | *Conexibacteraceae* | *Conexibacter* | 204 | (±16) | 334 | (±34) |
| 79* | *Actinobacteria* | *Solirubrobacterales* | *Conexibacteraceae* | *Conexibacter* | 751 | (±63) | 1102 | (±116) |
| 2285^d^* | *Actinobacteria* | *Actinobacteria* | *various* | *various* | 25 | (±5) | 74 | (±18) |
| 3095* | *Chloroflexi* | *Anaerolineales* | *Anaerolineaceae* | *uncl.Anaerolineaceae* | 26 | (±9) | 70 | (±24) |
| 2734* | *Chloroflexi* | *Thermomicrobia* | *uncl.Thermomicrobia* | *unclassified* | 18 | (±3) | 29 | (±5) |

**Table S2C: OTUs_0.25_** with significant abundance shifts between summer and winter

| **OTU#^a^** | **Consensus affiliation^b^** | |  | **Relative abundance (reads per sample)^c^** | | | | |
| --- | --- | --- | --- | --- | --- | --- | --- | --- |
|  | **Phylum/class** | **Order/group** | **Family** | **Genus** | **Summer** | | **Winter** | |
| *increased relative abundance in summer* | | |  |  |  |  |  |  |
| 64 | *Acidobacteria* | *various* | *various* | *various* | 1541 | (±351) | 1109 | (±238) |
| 102* | *Acidobacteria* | *Gp7* | *various* | *various* | 79 | (±13) | 51 | (±24) |
| *increased relative abundance in winter* | | |  |  |  |  |  |  |
| 20* | *Actinobacteria* | *Actinobacteria* | *various* | *various* | 5033 | (±628) | 6911 | (±926) |

^a^ Out of all OTUs_0.03_, OTUs_0.10_ and OTUs_0.25_ larger than 120 reads the table shows only those, which either consistently increased or decreased from summer to winter in all plots.

^b^Consensus of the affiliations of all reads in an OTU. “Unclassified” indicates that all reads in the OTU were unclassified at a given taxonomic level. “various” indicates that the individual reads in the OTU were affiliated with varying entries in the SILVA bacterial rRNA gene database

^c^ In total 17308 reads were analyzed from each sample, so that the number of reads affiliated with a given phylotype corresponds to the relative abundance of this phylotype. Means (±SE) are given and were calculated over the six samples obtained from the six different plots.

^d^ Data were square root transformed for the paired t-test.

* Significant difference between summer and winter: (paired t-test, n=6, *p<0.05, **p<0.01).

**Table S3. OTUs_0.03_ containing more than 600 reads ranked by their abundance**

| **OTU#** | ***Consensus affiliation*** | | | | ***Relative abundance (%)^a^*** | | | |
| --- | --- | --- | --- | --- | --- | --- | --- | --- |
|  | ***Phylum/class*** | ***Order/group*** | ***Family*** | ***Genus*** | ***Summer*** | | ***Winter*** | |
|  |  |  |  |  | ***Control*** | ***Warmed*** | ***Control*** | ***Warmed*** |
| 4571 | *α-Proteobacteria* | *Rhizobiales* | *Bradyrhizobiaceae* | *unclassified* | 4.1 (2.5) | 6.4 (±3.5) | 4.7 (±1.7) | 5.9 (±1.7) |
| 4543** | *α-Proteobacteria* | *Rhizobiales* | *Hyphomicrobiaceae* | *Rhodoplanes* | 2.7 (0.2) | 2.6 (±0.2) | 2.2 (±0.4) | 1.7 (±0.3) |
| 11804 | *Chloroflexi* | *unclassified* | *unclassified* | *unclassified* | 1.3 (0.3) | 1.1 (±0.1) | 1.9 (±0.5) | 1.3 (±0.1) |
| 317 | *α-Proteobacteria* | *unclassified* | *unclassified* | *unclassified* | 0.7 (0.4) | 1.5 (±0.7) | 1.8 (±0.2) | 1.6 (±0.1) |
| 3672 | *Actinobacteria* | *Actinomycetales* | *Acidothermaceae* | *Acidothermus* | 1.3 (0.4) | 1.4 (±0.7) | 1.3 (±0.2) | 1.2 (±0.3) |
| 1300 | *α-Proteobacteria* | *unclassified* | *unclassified* | *unclassified* | 0.4 (0.4) | 1.0 (±0.7) | 0.8 (±0.1) | 1.0 (±0.2) |
| 410** | *α-Proteobacteria* | *Rhizobiales* | *Hyphomicrobiaceae* | *unclassified* | 1.3 (0.3) | 1.1 (±0.4) | 0.3 (±0.0) | 0.5 (±0.2) |
| 1524 | *Nitrospira* | *Nitrospirales* | *Nitrospiraceae* | *Nitrospira* | 1.1 (0.4) | 0.6 (±0.3) | 0.6 (±0.2) | 0.5 (±0.2) |
| 4556 | *α-Proteobacteria* | *Rhizobiales* | *Hyphomicrobiaceae* | *Rhodoplanes* | 0.4 (0.3) | 0.8 (±0.4) | 0.4 (±0.2) | 1.0 (±0.1) |
| 9730 | *Actinobacteria* | *Actinomycetales* | *Microbacteriaceae* | *Agromyces* | 0.3 (0.2) | 0.5 (±0.0) | 1.3 (±0.7) | 0.4 (±0.1) |
| 476 | *α-Proteobacteria* | *Rhizobiales* | *Hyphomicrobiaceae* | *Pedomicrobium* | 0.8 (0.1) | 0.9 (±0.2) | 0.4 (±0.1) | 0.4 (±0.1) |
| 9857 | *Actinobacteria* | *Actinomycetales* | *Microbacteriaceae* | *Agromyces* | 0.7 (0.3) | 0.3 (±0.1) | 0.9 (±0.3) | 0.5 (±0.4) |
| 10535 | *Actinobacteria* | *Actinomycetales* | *Nocardioidaceae* | *Kribbella* | 0.4 (0.2) | 0.6 (±0.0) | 0.7 (±0.2) | 0.6 (±0.3) |
| 1042* | *α-Proteobacteria* | *Rhizobiales* | *Phyllobacteriaceae* | *Mesorhizobium* | 0.8 (0.2) | 0.7 (±0.1) | 0.5 (±0.1) | 0.3 (±0.1) |
| 4911 | *α-Proteobacteria* | *Rhizobiales* | *Bradyrhizobiaceae* | *Afipia* | 0.4 (0.4) | 0.7 (±0.2) | 0.5 (±0.2) | 0.5 (±0.1) |
| 7850 | *Actinobacteria* | *Solirubrobacterales* | *Conexibacteraceae* | *Conexibacter* | 0.6 (0.2) | 0.4 (±0.2) | 0.6 (±0.2) | 0.4 (±0.1) |
| 211 | *Actinobacteria* | *Solirubrobacterales* | *Conexibacteraceae* | *Conexibacter* | 0.7 (0.2) | 0.4 (±0.2) | 0.5 (±0.1) | 0.3 (±0.1) |
| 7295 | *Actinobacteria* | *Actinomycetales* | *unclassified* | *unclassified* | 0.2 (0.1) | 0.4 (±0.1) | 0.3 (±0.1) | 1.0 (±0.4) |
| 202* | *Actinobacteria* | *Solirubrobacterales* | *Conexibacteraceae* | *Conexibacter* | 0.4 (0.0) | 0.3 (±0.1) | 0.5 (±0.1) | 0.5 (±0.1) |
| 190 | *Actinobacteria* | *Solirubrobacterales* | *Conexibacteraceae* | *Conexibacter* | 0.4 (0.0) | 0.3 (±0.1) | 0.5 (±0.2) | 0.6 (±0.1) |
| 10463 | *Actinobacteria* | *Actinomycetales* | *Nocardioidaceae* | *Kribbella* | 0.3 (0.2) | 0.5 (±0.1) | 0.4 (±0.1) | 0.4 (±0.2) |
| 4919 | *α-Proteobacteria* | *Rhizobiales* | *unclassified* | *unclassified* | 0.2 (0.2) | 0.3 (±0.3) | 0.5 (±0.4) | 0.5 (±0.2) |
| 7926* | *Actinobacteria* | *Solirubrobacterales* | *Conexibacteraceae* | *Conexibacter* | 0.1 (0.1) | 0.3 (±0.1) | 0.3 (±0.1) | 0.7 (±0.1) |
| 9721 | *Actinobacteria* | *Actinomycetales* | *Microbacteriaceae* | *Agromyces* | 0.3 (0.1) | 0.3 (±0.1) | 0.5 (±0.1) | 0.4 (±0.2) |
| 7305 | *Actinobacteria* | *Solirubrobacterales* | *Conexibacteraceae* | *Conexibacter* | 0.4 (0.1) | 0.3 (±0.1) | 0.4 (±0.1) | 0.3 (±0.2) |
| 4613 | *α-Proteobacteria* | *Rhizobiales* | *Hyphomicrobiaceae* | *Rhodoplanes* | 0.5 (0.1) | 0.3 (±0.1) | 0.3 (±0.2) | 0.2 (±0.1) |
| 163 | *α-Proteobacteria* | *Rhizobiales* | *Hyphomicrobiaceae* | *Rhodoplanes* | 0.1 (0.1) | 0.4 (±0.2) | 0.2 (±0.1) | 0.5 (±0.1) |
| 9547 | *Actinobacteria* | *Actinomycetales* | *Propionibacteriaceae* | *unclassified* | 0.5 (0.2) | 0.2 (±0.1) | 0.3 (±0.2) | 0.2 (±0.0) |
| *6925* | *unclassified* | *unclassified* | *unclassified* | *unclassified* | 0.4 (0.0) | 0.3 (±0.1) | 0.2 (±0.0) | 0.3 (±0.1) |
| 9812 | *Actinobacteria* | *Actinomycetales* | *unclassified* | *unclassified* | 0.3 (0.1) | 0.3 (±0.1) | 0.3 (±0.1) | 0.3 (±0.1) |
| 8010 | *Actinobacteria* | *Solirubrobacterales* | *Conexibacteraceae* | *Conexibacter* | 0.1 (0.1) | 0.3 (±0.1) | 0.3 (±0.1) | 0.5 (±0.1) |

^a^Abundances are expressed as percentage of the total 17308 reads analyzed per sample (mean±standard error, n=3). Together the OTUs listed in this table accounted for 24% of all reads.

*Significant difference in abundance between summer and winter: *p<0.05, **p<0.01

A
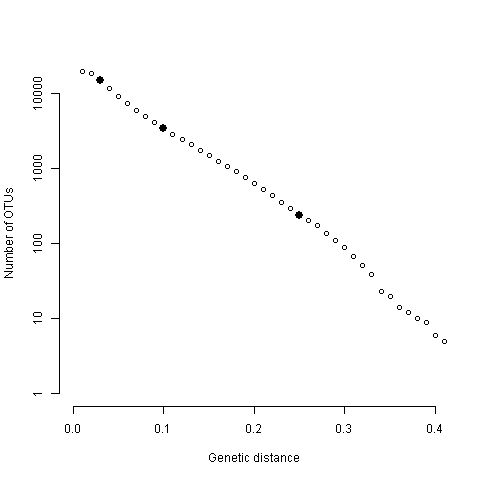
 B
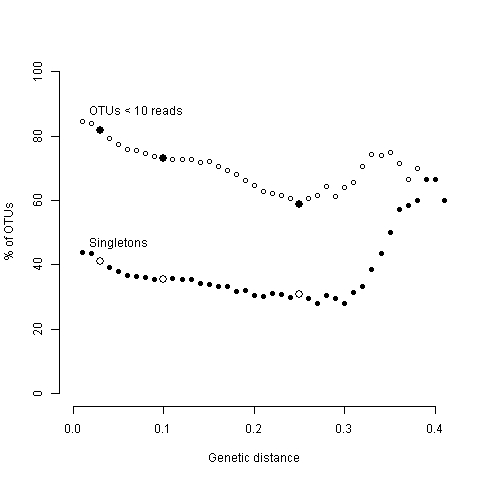


C
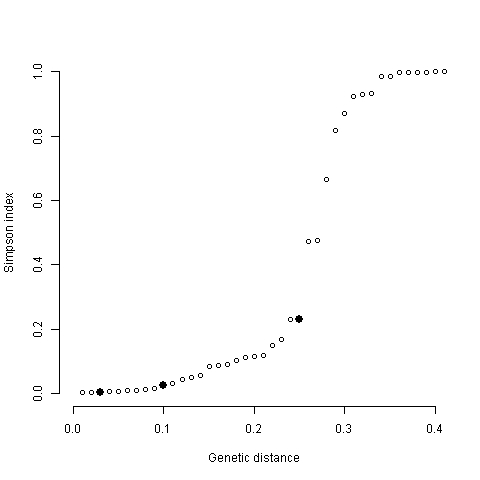
 D
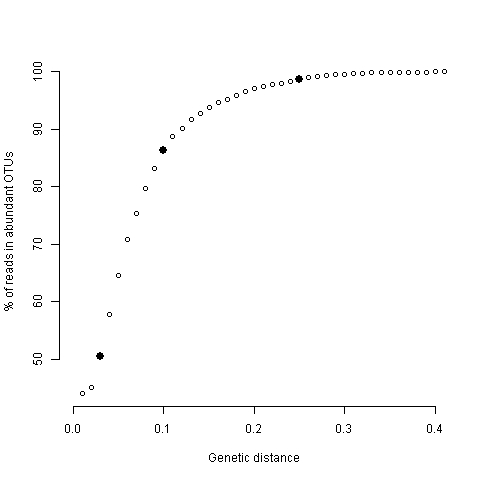


E
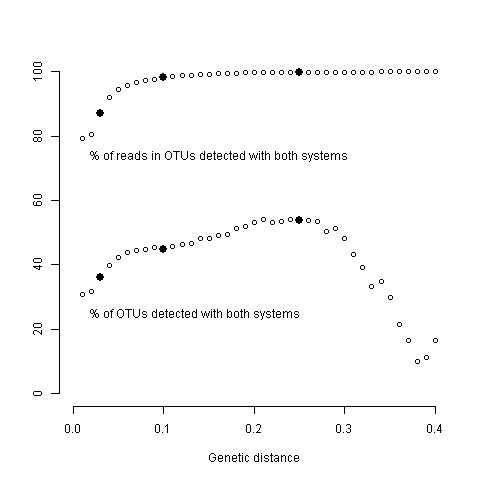


**Figure S1.** **Clustering of reads into OTUs at different genetic distances.** A) Number of OTUs, B) proportion of singletons and OTUs < 10 reads in total OTUs, C) Simpson index indicating the probability of two reads to be in the same OTU and D) proportion of reads in OTUs containing ≥ 120 reads. E) OTUs containing reads with primer 27f at read start as well as reads with primer 518r at read start. The genetic distances of 0.03, 0.10 and 0.25 are highlighted in each panel.

**Figure S2. Phylogenetic community composition of libraries obtained with primers 27f and 518r at read start.** Relative abundance of A) abundant phyla (>1% of total reads) and proteobacterial classes, B) rare phyla. Phyla with a significantly higher proportion in libraries obtained with 27f at read start are labeled with asterisks (*p<0.05, ** p<0.01, paired t-Test, n=12). Phyla with a significantly higher proportion in libraries obtained with 518r at read start are apostrophed (‘p<0.05, “ p<0.01, paired t-Test, n=12). ^a^ data were square root transformed for the paired t-Test. Sample symbols: ● ■ ▲- control plots, ○ ◊ - warmed plots, red-summer, blue-winter.

**Figure S3.** **OTU counts and estimates of richness and diversity of the 24 individual amplicon libraries.** A-C) Libraries with primer 27f at read start. D-F) libraries with primer 518r at read start. A and D) OTUs_0.03_, B and E) OTUs_0.10_, C and F) OTUs_0.25_. Sample symbols: ● ■ ▲- control plots, ○ ◊ - warmed plots, black-summer, **grey-winter**. Observed OTUs and ACEs are plotted on the primary, 1/D-values on the secondary Y-axis. Error bars span the 95% confidence intervals.


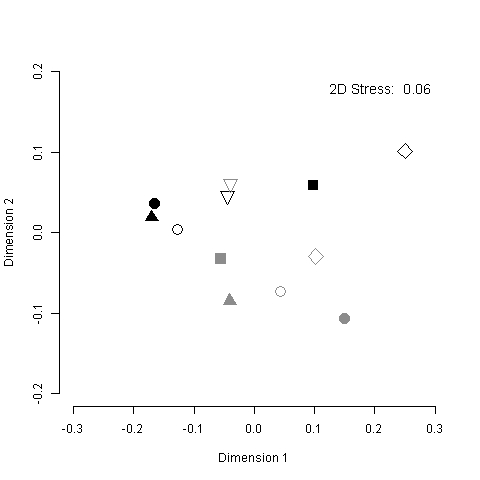


**Figure S4. NMDS of weighted Unifrac distances between samples.**

Sample symbols: ● ■ ▲- control plots, ○ ◊ - warmed plots, black-summer, grey-winter.
